# The Influence of Sevelamer Hydrochloride and Calcium Carbonate on Markers of Inflammation and Oxidative Stress in Hemodialysis at Six Months of Follow-Up

**DOI:** 10.3389/fmed.2021.714205

**Published:** 2021-11-25

**Authors:** Elodia Nataly Díaz-De la Cruz, José Ignacio Cerrillos-Gutiérrez, Andrés García-Sánchez, Carlos Gerardo Prado-Nevárez, Jorge Andrade-Sierra, Basilio Jalomo-Martínez, Adriana Banda-López, Enrique Rojas-Campos, Alejandra Guillermina Miranda-Díaz

**Affiliations:** ^1^Department of Physiology, University Health Sciences Center, Universidad de Guadalajara, Guadalajara, Mexico; ^2^Department of Nephrology and Organ Transplant Unit, National Western Medical Centre, Mexican Institute of Social Security, Specialties Hospital, Guadalajara, Mexico

**Keywords:** oxidative stress markers, ESRD, calcium carbonate, antioxidants, sevelamer hydrochloride

## Abstract

Patients with end-stage renal disease (ESRD) present alterations in mineral and bone metabolism. Hyperphosphatemia in ESRD is considered an independent risk factor for cardiovascular disease (CVD), increasing morbidity, and mortality. Sevelamer hydrochloride is a calcium-free, non-absorbable phosphate-chelating polymer. Calcium carbonate chelator is helpful in controlling serum phosphate levels. There is insufficient information on the influence of sevelamer hydrochloride and calcium carbonate on the behavior of oxidative stress (OS) markers and inflammation in patients on hemodialysis (HD). A randomized open clinical trial was carried out on patients to evaluate sevelamer hydrochloride and calcium carbonate influence at 6 months of study follow-up. Levels of oxidants (LPO, NO, and 8-isoprostanes), antioxidants (SOD and TAC), oxidative DNA damage (8-OHdG and hOGG1), pro-inflammatory cytokines (IL-6 and TNF-α), and inflammation markers (ferritin and C-reactive protein) were measured with colorimetric and ELISA methods. We found a significant increase in oxidants LPO and NO, and antioxidants SOD and TAC, and downregulation of IL-6 and TNF-α. Ferritin decrease at 6 months follow-up in the sevelamer hydrochloride group. Increase in C-reactive protein was found in the group of patients treated with calcium carbonate. In conclusion, we found an oxidative state imbalance with increase in LPO and NO oxidants. The activity of the antioxidant enzymes (SOD and TAC) was also found to increase, suggesting a compensatory effect in the face of increase in oxidants. The same phenomenon was observed with increase in the oxidative damage marker to DNA and the increase in the DNA repair enzyme, suggesting a compensatory effect. Pro-inflammatory cytokines were predominantly downregulated by TNF-α in the group that ingested sevelamer hydrochloride in the final determination at 6 months of follow-up. Serum ferritin levels decreased significantly at the end of follow-up in patients on HD in the sevelamer hydrochloride group. The management of hyperphosphatemia with sevelamer hydrochloride appears to have obvious anti-inflammatory and antioxidant benefits.

## Introduction

Disorders of mineral and bone metabolism are frequent complications in patients with end-stage renal disease (ESRD). Hyperphosphatemia is an inevitable consequence of ESRD, the presence of which is associated with increased morbidity and mortality (1). Oxidative stress (OS) is characterized by an imbalance between decrease in antioxidant defense mechanisms and increase in oxidant products. OS appears in early stages of chronic kidney disease (CKD), progresses along with worsening kidney failure, and is aggravated by the hemodialysis (HD) process. Observational studies have reported that hyperphosphatemia is an independent risk factor for cardiovascular disease (CVD) capable of increased mortality in patients on dialysis (2). Control of serum phosphorus (Pi) is key to improving clinical outcomes for patients on dialysis. The predominant drug treatment for hyperphosphatemia has been calcium-based phosphate binders (calcium carbonate or calcium acetate). However, calcium salts are related to arterial calcification (3). New phosphate binders have made it possible to dispense with calcium in the formulation (4). Sevelamer hydrochloride is a non-absorbable, calcium-free phosphate-chelating polymer that has been available since 1998 (5). Sevelamer hydrochloride is an anion exchange resin, allowing it to exchange negatively charged ions. Sevelamer hydrochloride has a high affinity for phosphate ions and exerts its therapeutic action in the intestine, exchanging chloride for phosphate and forming an insoluble compound that is excreted in feces (6).

**Figure 1:**
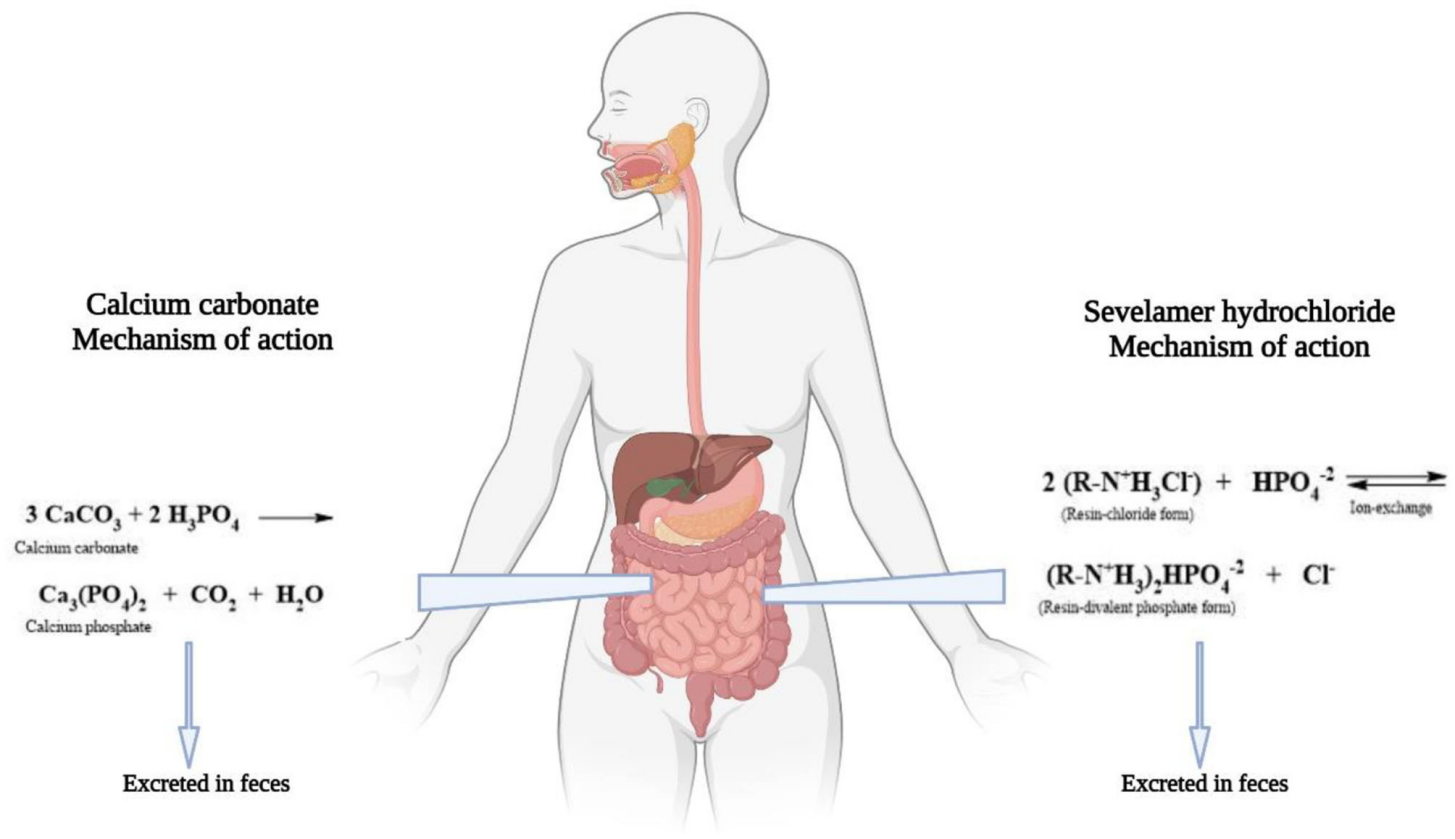
Sevelamer hdyrochloride and calcium carbonate mechanism. Binding of sevelamer hydrochloride and calcium carbonate with phosphate. Right side shows the ion exchange between the resin and the phosphate. Left side shows the reaction between calcium carbonate and phosphate.

Figure 1 briefly shows the binding mechanism of sevelamer hydrochloride and calcium carbonate with ingested Pi. Some studies have reported potential benefits of the use of sevelamer hydrochloride. However, there are insufficient data to establish the superiority of phosphate binders without calcium content over those containing calcium with CVD development (7). Sevelamer hydrochloride helps prevent calcium overload, reduces hyperphosphatemia, and translates into better clinical results. Sevelamer hydrochloride is associated with better survival in patients on HD (8). Experimental, observational, and clinical trials have shown sevelamer hydrochloride to have pleiotropic effects beyond the control of hyperphosphatemia. Sevelamer hydrochloride acts on inflammation, OS, lipid profile, atherogenesis, vascular calcification, and endothelial dysfunction, and reduces uremic toxins (9, 10). Patients on HD have a high risk of mortality from all causes and cardiovascular events. Excessive OS and chronic inflammation emerge as new contributors of accelerated atherosclerosis and elevated mortality in ESRD. Patients on HD show an increase in free radicals due to retention of uremic toxins, reduction in antioxidants, activation of leukocytes, infectious processes, and presence of comorbidities (11). The potential benefits of sevelamer hydrochloride to decrease inflammation and OS markers need to be studied (12).

The purpose of the study was to evaluate the influence of sevelamer hydrochloride and calcium carbonate on OS markers and inflammation in patients on HD at 6 months of follow-up.

## Patients and Methods

### Patients

A randomized open clinical trial was carried out on patients to evaluate sevelamer hydrochloride and calcium carbonate influence at 6 months of study follow-up. An open randomized clinical trial was designated, because it was not possible to mask the drugs. The medications were obtained through a prescription that was exchanged at the pharmacy by the patient himself. The initial doses of sevelamer hydrochloride were two tablets of 800 mg every 12 h. It was necessary to adjust the dose (±1 tablet) each month to achieve a target serum Pi of 5 mg/dl and considering drug tolerance. Serum Pi was monitored each month to determine the need for dose adjustment using a colorimetric method for its determination. The doses administered at the end of the study were 1,600, 2,400, and 3,200 mg. The titration of the drug was gradual and according to each patient requirement (13).

Randomization and treatment allocation were based on simple randomization using the Microsoft Excel software that generates random numbers. Male and female patients aged 18–60 years were included. Medications were administered for comorbidities, for arterial hypertension (nifedipine, metoprolol, losartan), for DM (insulin), for anemia (folic acid, erythropoietin). The diet of ESRD patients includes limiting fluids, consuming a low-protein diet, and reducing their intake of salt, potassium, Pi, and other electrolytes. In ESRD patients it is important to consume enough calories to avoid losing weight. No dietary supplements were administered. All patients who have ESRD, with GFR < 10 ml/min/1.73 m^2^, were subjected to three HD sessions per week with the same type of filter without the possibility of determining albuminuria. The patients were attended in the HD Unit of the Department of Nephrology of the High Specialty Medical Unit of the Mexican Institute of Social Security (IMSS) in Guadalajara, Jalisco, Mexico. Patients who ingested antioxidants (vitamins E and C) 3 months before the study and patients with clinical or biochemical data of an infectious process were not included. Patients who withdrew informed consent, with <80% adherence to treatment and patients with adverse drug events were excluded from the study.

### Data Collection

Sixty-four patients were enrolled, and two intervention groups with 32 patients were formed. One group was composed of patients treated with sevelamer hydrochloride, and the other was made up of patients treated with calcium carbonate. Treatment adherence was verified by the investigator in charge of prescribing the drug to each patient. Adherence was determined by recording the drug supply of a pharmacy according to the stipulated time and loaded into the file of each patient. Body mass index (BMI) and treatment group were recorded. Biochemical data were obtained the first day of treatment immediately before starting HD and at 6 months of follow-up: hemoglobin, urea, uric acid, cholesterol, triglycerides, albumin, urea, serum creatinine, and uric acid, serum electrolytes (sodium, potassium, chlorine, Pi, calcium, and magnesium), ferritin, iron saturation, bicarbonate, C-reactive protein (CRP), vitamin D, pH, and parathyroid hormone (PTH). Venous blood samples, 5 ml, were collected in a dry tube and 5 ml in a tube with 7.2 mg of dipotassium ethylene-diamine-tetra-acetic acid (K2 EDTA). The samples were centrifuged at 3,500 rpm for 10 min to obtain serum and plasma. Six months after starting treatment (sevelamer hydrochloride or calcium carbonate), blood samples were collected to compare the results. The aliquots obtained were stored at −80°C to analyze later inflammation markers, oxidants, antioxidants, and oxidative damage to DNA.

### OS Markers

#### Lipoperoxidation (LPO) Products

The levels of LPO in plasma were measured using an FR22 assay kit (Oxford Biomedical Research Inc., Oxford, MI, United States) and according to the instructions of the manufacturer.

#### 8-iso Prostaglandin F_2α_ 8-Isoprostanes (8-IP)

An immunoassay reagent kit from Abcam Company® (Cambridge, England) was used according to the instructions of the manufacturer.

#### Nitric Oxide (NO)

Before determining NO levels, the serum samples were deproteinized by adding 6 mg of zinc sulfate to 400 μl of the sample vortex for 1 min, and the samples were centrifuged at 10,000 × g for 10 min at 4°C. The determination of NO was carried out per the kit (NB98; Oxford Biochemical Research Inc., Oxford, MI, United States).

### Antioxidants

#### Superoxide Dismutase (SOD)

The instructions of the kit manufacturer (SOD No. 706002; Cayman Chemical Company®, Ann Arbor, Michigan, USA) were followed to measure SOD activity. The levels are reported in U/ml.

#### Total Antioxidant Capacity (TAC)

Evaluations of total antioxidant capacity (TAC) were performed according to the instructions of the kit manufacturer (Total Antioxidant Power Kit, No. TA02.090130; Oxford Biomedical Research®, Oxford, MI, United States).

#### Marker of Oxidative DNA Damage

The serum levels of 8-hydroxy-2′-deoxyguanosine (8-OHdG) were measured using an 8-hydroxy-2-deoxyguanosine ELISA kit ab201734 (Abcam®, Cambridge, MA, United States).

#### DNA Repair Enzyme—Oxoguanine Glycosylase

It was performed by the manufacturer of human 8-oxoguanine DNA glycosidase (hOGG1) ELISA kit no. MBS702793, MyBioSource® (San Diego, USA).

### Pro-inflammatory Cytokines

#### TNF-α and IL-6

The sandwich ELISA method of the commercial 900-K25 and 900-K16 kit suggested by the manufacturer Peprotech (New Jersey, USA) was followed. The absorbances of the wells were recorded at 405 nm with a correction of 650 nm.

#### Ferritin and C-Reactive Protein (CRP)

They were determined by immuno-turbidimetry. Antibody-coated latex particles were agglutinated by ferritin or CRP present in the patient sample. The agglutination process caused an absorbance change proportional to the concentration of the inflammation marker in the sample, and the concentration in the sample was determined by comparison with a standard.

### Statistical Analysis

Sample size calculation was performed by comparing two means formula (14). In the available literature, we did not find longitudinal studies on the effect of sevelamer on OS markers. Therefore, we used as a reference the variation of TNF-α in patients on HD treated with sevelamer reported by Peres et al. (13). There were no losses from the included population. The patients underwent HD three times a week with the same type of filter; no intention-to-treat analysis was required. The results are presented in measures of central tendency and dispersion for quantitative variables. Qualitative variables are shown in frequencies and percentages. The Kolmogorov–Smirnov test was performed to determine the normality of the data. For intra-group differences, the Wilcoxon rank test was performed according to the distribution obtained. Mann–Whitney *U*-test or Student's *t*-test was performed on independent samples for treatment inter-group differences. ANOVA or Kruskal-Wallis test was performed for dose inter-group differences. All results with a value of *p* ≤ 0.05 were documented as statistically significant.

### Ethical Considerations

The study was followed under the ethical principles for medical research in human beings stipulated in the declaration of Helsinki 64th General Assembly, Fortaleza Brazil, October 2013, and the Standards of Good Clinical Practice according to the guidelines of the International Conference on Harmonization. Under the General Health Law's provisions following the Regulations of the General Health Law on Research for Health Article 17 of Mexico, the study corresponds to category III. All the patients signed the Low Information Consent in the presence of witnesses. The study was evaluated and approved by the Local Ethics and Research Committee at the *Centro Médico Nacional de Occidente*, Instituto Mexicano del Seguro Social (R-2021-1301-063).

## Results

Sixty-four patients were included, 32 in the sevelamer hydrochloride group and 32 patients in the calcium carbonate group. Thirty-eight female and 26 male patients were included. Body weight was similar in both groups, 64.79 ± 19.1 kg of patients treated with sevelamer hydrochloride and 63.55 ± 22.46 kg of those treated with calcium carbonate. The time in the HD program was also similar, 8.06 ± 4.78 years in the sevelamer hydrochloride group and 7.05 ± 5.06 years in the group treated with calcium carbonate. In the sevelamer hydrochloride group, a normal BMI was found in 64.5% of the patients, 12.9% were overweight, and 22.6% were obese. In the calcium carbonate group, the BMI was normal in 71.4%, 14% of the patients were overweight, and 14.3% were obese. 74.2% of the patients in the sevelamer hydrochloride group and 71.4% of the patients treated with calcium carbonate had diabetes mellitus (DM). 32.3% of the patients treated with sevelamer hydrochloride and 19% of those treated with calcium carbonate had arterial hypertension (AH) (Table 1).

**Table 1:** Baseline anthropometric data.

	**Sevelamer hydrochloride**	**Calcium carbonate**	***P***
**Gender (%)**			
Male	41.90	33.3	0.61
Female	58.1	66.6	
**Weight** (kg)	64.79 ± 19.10	63.55 ± 22.46	0.8
**BMI** (Kg/m^2^)	24.95 ± 5.93	24.02 ± 5.28	0.72
**BMI (%)**			
Normal	64.5	71.4	0.44
Overweight	12.9	14	
Obesity	22.6	14.3	
**Time in HD** (years)	8.06 ± 4.78	7.05 ± 5.06	0.038^¥^
**DM (%)**			
Yes	74.2	71.4	0.11
No	25.8	28.6	
**AH (%)**			
Yes	32.3	19	0.31
No	67.7	81	

*Values are expressed as mean ± standard deviation or percentages*.

¥ *Chi^2^ test. BMI, Body mass index; DM, Diabetes Mellitus; AH, Arterial Hypertension*.

Table 2 shows the causes of kidney failure in both treatment groups. The unknown cause is highlighted.

**Table 2:** ESRD causes.

	**Sevelamer hydrochloride**	**Calcium carbonate**
	**n- 32**	**n-32**
Unknown	23	22
DM2	5	4
Polycystic kidney disease	1	1
Systemic lupus erythematous	1	2
Nephrolithiasis	1	–
Other	1	3

### Biochemical Data

The baseline hemoglobin levels in both groups were similar, 10.5 ± 1.63 mg/dl in the sevelamer hydrochloride group and 10.14 ± 2.23 mg/dl in the calcium carbonate group. In the determination at six months of follow-up, the hemoglobin levels in the sevelamer hydrochloride group increased to 12.15 ± 1.93 mg/dl (*p* = 0.001) without observing changes in those who received calcium carbonate. 10.85 ± 2.57 mg/dl (*p* = 0.23). Ferritin levels decreased significantly in the sevelamer hydrochloride group, 201.67 ± 60.39 ng/ml with respect to the 396.24 ± 709.77 ng/ml in the calcium carbonate group (*p* = 0.004). CRP levels showed an increase in both groups (Table 3).

**Table 3:** Sevelamer hydrochloride or Calcium carbonate dose.

	**Sevelamer hydrochloride**		**WCX**	**Calcium carbonate**		**WCX**	**U-MW**
	**n-32**			**n-32**			
	**Baseline**	**Six-months**	***p***	**Baseline**	**Six-months**	***p***	***p***
**Oxidants**							
LPO (mM)	15.70 ± 3.78	35.85 ± 6.31	0.022^**^	12.92 ± 2.03	27.53 ± 8.31	0.11	0.68
NO (μg/mL)	1196.01 ± 112.08	1387.61 ± 175.97	<0.001^**^	1215.70 ± 163.65	1322.02 ± 200.54	<0.001^**^	0.8
8-IP (pg/mL)	50.46 ± 10.20	73.11 ± 13.34	0.07	112.82 ± 14.62	90.32 ± 9.08	0.12	<0.001^*^
**Markers of oxidative damage to DNA**							
8-OHdG (ng/mL)	6.29 ± 2.22	9.52 ± 7.21	0.033^**^	8.60 ± 4.77	8.88 ± 5.43	0.84	0.69
hOGG1 (pg/mL)	3.67 ± 0.53	21.84 ± 6.54	<0.001^**^	7.89 ± 1.52	14.68 ± 4.86	0.22	0.10
**Antioxidants**							
SOD (U/L)	4.75 ± 3.07	7.69 ± 3.19	<0.001^**^	6.03 ± 4.39	8.78 ± 5.43	0.016^**^	0.51
TAC (μM)	0.62 ± 0.19	0.82 ± 0.22	<0.001^**^	0.69 ± 0.21	0.87 ± 0.34	<0.001^**^	0.86
**Pro-inflammatory cytokines**							
IL-6 (pg/mL)	3.00 ± 1.86	2.41 ± 0.92	0.37	3.49 ± 1.66	2.70 ± 1.19	0.037^**^	0.38
TNF- α (pg/mL)	2.44 ± 1.92	2.01 ± 0.47	0.003^**^	2.12 ± 0.73	1.75 ± 0.53	0.009^**^	0.97
Ferritin (ng/mL)	234.63 ± 404.33	201.67 ± 60.39	0.004^**^	158.44 ± 153.60	396.24 ± 709.77	0.59	0.10
CRP (mg/L)	7.04 ± 7.34	11.65 ± 10.55	0.08	7.08 ± 9.39	9.53 ± 4.10	0.043^**^	0.65
**Biochemical data**							
Hb (mg/dL)	10.50 ± 1.63	12.15 ± 1.93	0.001^**^	10.14 ± 2.23	10.85 ± 2.57	0.26	0.23
Urea (mg/dL)	108.43 ± 37.93	102.80 ± 32.17	0.42	130.27 ± 43.11	128.91 ± 30.14	0.26	0.69
Uric acid (μmol/L)	5.16 ± 1.48	5.18 ± 1.19	0.79	5.27 ± 1.94	5.15 ± 1.67	0.6	0.76
SCr (mg/dL)	10.56 ± 2.34	10.41 ± 3.1	0.6	10.01 ± 3.2	9.51 ± 3.2	0.6	0.1
Na (mmol/L)	138.79 ± 2.29	136.95 ± 0.96	0.008 ^**^	138.65 ± 2.55	138.09 ± 2.63	0.49	0.75
K (mmol/L)	5.17 ± 0.52	5.15 ± 0.59	0.38	5.19 ± 1.13	5.51 ± 0.77	0.60	**0.048**
Pi (mmol/L)	6.29 ± 1.61	5.71 ± 52	0.040^**^	5.81 ± 2.39	6.15 ± 1.60	0.59	0.79
Ca (mg/mL)	8.86 ± 1.14	8.93 ± 1.02	0.63	8.71 ± 1.43	8.05 ± 2.34	0.44	0.22
HCO_3_ (mEq/L)	23.62 ± 3.16	22.14 ± 4.90	0.79	21.62 ± 4.49	23.41 ± 2.74	0.047^**^	<0.001^**^

*Values are expressed as mean ± standard deviation or standard error of the mean*.

* *Mann-Whitney U-test*.

** *Wilcoxon rank test. LPO, Lipoperoxides; NO, Nitric Oxide; 8-IP, 8-isoprostanes; 8-OHdG, 8-hydroxy-2'-deoxyguanosine; hOGG1, Oxoguanine glycosylase; SOD, Superoxide dismutase; TAC, Total Antioxidant Capacity; IL-6, Interleukin 6; TNF-α, Tumor necrosis factor alpha; Hb, hemoglobin; Na, Sodium; K, Potassium; Pi, Phosphate; CRP, C-reactive protein; Vit. D, vitamin D; HCO3, Bicarbonate. Data in bold are statistically significant p-values*.

### OS Markers

#### LPO, 8-IP, NO

The LPO levels increased significantly between the baseline levels 15.7 ± 3.79 mM and the final determination 35.85 ± 6.32 mM (*p* = 0.022) in the sevelamer hydrochloride group. The same behavior was observed in basal NO levels 1,196.01 ± 112.08 μg/ml compared with the increase found at the end of follow-up, 1,387.61 ± 175.97 μg/ml (*p* = 0.001). The group treated with calcium carbonate had similar results between the baseline and 6 months of treatment. The 8-IP levels in the sevelamer hydrochloride group were 50.46 ± 10.2 vs. 73.11 ± 13.34 pg/ml (*p* = 0.07) (Table 3).

#### Antioxidants

The activity of the SOD enzyme was found to be significantly increased in the final determination of those treated with sevelamer hydrochloride, 7.69 ± 3.19 U/L (*p* < 0.001) and with calcium carbonate, 8.78 ± 5.43 U/L (*p* = 0.016). The levels of TAC were significant increased at the end of the follow-up for the sevelamer hydrochloride (0.82 ± 0.22 μM, *p* < 0.001) and calcium carbonate, (0.87 ± 0.34 μM, *p* < 0.001) groups (Table 3).

#### Pro-inflammatory Cytokines

We found a significant decrease in the expression of the pro-inflammatory cytokine TNF-α between baseline levels, 2.44 ± 1.92 pg/ml, and the final result, 2.01 ± 1.47 pg/ml (*p* = 0.003), in patients treated with sevelamer hydrochloride. The same behavior was observed in those who received calcium carbonate between the expression of basal TNF-α, 2.12 ± 0.73 pg/ml, and final result, 1.75 ± 0.53 pg/ml (*p* = 0.009). Ferritin levels were decreased at the end of follow-up 201.67 ± 60.39 ng/ml (*p* = 0.04), in the patients who ingested sevelamer hydrochloride. CRP levels were increased in the patients treated with calcium carbonate, 10.66 ± 6.07 mg/L (*p* = 0.008) (Table 3).

#### Oxidative Damage to DNA

The expression of the oxidative DNA damage marker (8-OHdG) in the final determination increased, 9.52 ± 7.21 ng/ml (*p* = 0.033), compared with the baseline determination of 6.29 ± 2.22 ng/ml in the hydrochloride sevelamer group. The same behavior was observed with the DNA repair enzyme in the final determination, 21.84 ± 6.54 pg/ml (*p* < 0.001), of the patients treated with sevelamer hydrochloride compared with the baseline value of 3.67 ± 0.53 pg/ml. The group that ingested calcium carbonate did not change the levels between the baseline and final determination (Table 3).

### Hemodialysis and Sevelamer Hydrochloride Dose

Three daily doses (1,600, 2,400, and 3,200 mg) of sevelamer hydrochloride were administered to the patients in the HD program. Patients who ingested 3,200 mg had a significant increase in LPO in the final determination of the study, 48.85 ± 42.79 mM (*p* = 0.011), compared with the baseline level of 9.18 ± 4.52 mM. NO levels were also increased in the final determination in patients who received 1,600 mg, 1,372.14 ± 177.5 μg/ml (*p* = 0.031), and 3,200 mg, 1,319.25 ± 158.35 μg/ml (*p* = 0.028). The DNA repair enzyme was found to be significantly increased in the final determination in patients who ingested 1,600 mg of sevelamer hydrochloride 17.74 ± 9.02 (*p* = 0.028).

The activity of the antioxidant enzyme SOD was significantly increased at 6 months of follow-up only in patients who ingested 1,600 mg of sevelamer hydrochloride 0.91 ± 0.25 μM (*p* = 0.047). TAC levels were found to be increased in the final determination in patients who ingested 1,600 mg, 0.91 ± 0.25 μM, (*p* = 0.004), and 3,200 mg, 0.85 ± 0.28 μM (*p* = 0.011) of sevelamer hydrochloride.

The expression of IL-6 decreased significantly in the final determination in those who ingested 1,600 mg, 2.3 ± 1.01 pg/ml (*p* = 0.03). The expression of TNF-α decreased at the end of the study in those who ingested 3,200 mg, 1.8 ± 0.54 pg/ml (*p* = 0.038). Hemoglobin significantly improved in the final determination in patients who ingested 1,600 mg, 12.13 ± 2.81 mg/day (*p* = 0.012), and 3,200 mg, 11.2 ± 1.63 mg/dl (*p* = 0.03) of sevelamer hydrochloride. Ferritin levels decreased significantly at the end of the study in those who ingested 1,600 mg, 190.99 ± 282.23 ng/ml, (*p* = 0.028), and 3,200 mg, 231.47 ± 154.03 ng/ml (*p* = 0.05) of sevelamer hydrochloride. The levels of CRP, 9.63 ± 7.36 mg/L (*p* = 0.043), and vitamin D, 28.7 ± 12.65 nmol/L (*p* = 0.018), increased at the end of the study in patients who ingested 1,600 mg of sevelamer hydrochloride (Table 4).

**Table 4:** Doses of sevelamer hydrochloride intervention Calcium carbonate dose.

	**1,600 mg**		**WCX**	**2,400 mg**		**WCX**	**3,200 mg**		**WCX**	**K-W**
	**n-16**			**n-5**			**n-11**			
	**Baseline**	**Six-months**	***p***	**Baseline**	**Six-months**	***p***	**Baseline**	**Six-months**	***p***	***p***
**Oxidants**										
LPO (mM)	14.48 ± 9.25	37.36 ± 36.51	0.12	24.20 ± 17.55	11.43 ± 4.02	0.65	9.18 ± 4.52	48.85 ± 42.79	**0.011**	0.54
NO (μg/mL)	1,209.88 ± 92.41	1,372.14 ± 177.50	**0.031**	1,301.14 ± 134.41	1,383.12 ± 272.53	0.65	1,120.51 ± 150.09	1,319.25 ± 158.35	**0.028**	0.89
8-IP (pg/mL)	51.96 ± 31.15	89.33 ± 84.95	0.16	18.41	55.78	—	53.28 ± 35.85	65.53 ± 34.06	0.51	0.90
**Markers of oxidative damage to DNA**										
8-OHdG (ng/mL)	7.32 ± 4.90	8.06 ± 3.64	0.57	7.65	7.33	—	6.23 ± 1.74	7.01 ± 2.71	0.37	0.48
hOGG1 (ng/mL)	3.33 ± 0.73	19.61 ± 8.80	**0.017**	7.95	14.5	—	3.25 ± 0.32	14.31 ± 10.24	0.051	0.29
**Antioxidants**										
SOD (U/L)	5.00 ± 3.04	7.93 ± 4.35	**0.047**	6.24 ± 5.53	3.70 ± 2.43	0.65	5.42 ± 4.40	8.18 ± 4.06	0.16	0.89
TAC (μM)	0.71 ± 0.17	0.91 ± 0.25	**0.004**	0.73 ± 0.00	0.69 ± 0.12	0.65	0.53 ± 0.20	0.85 ± 0.28	**0.011**	0.49
**Pro-inflammatory cytokines**										
IL-6 (pg/mL)	3.24 ± 1.79	2.30 ± 1.01	**0.030**	1.91	2.47	—	3.05 ± 1.97	2.63 ± 0.91	0.95	0.42
TNF-α (pg/mL)	3.25 ± 2.83	2.70 ± 2.14	0.09	1.12	1.41	—	2.28 ± 0.91	1.80 ± 0.54	**0.038**	0.95
Ferritin (ng/mL)	201.58 ± 242.00	190.99 ± 282.23	**0.028**	21.15 ± 0.49	11.45 ± 2.05	0.18	378.36 ± 220.96	231.47 ± 154.03	0.050^**^	0.24
CRP (mg/L)	6.47 ± 6.87	9.63 ± 7.36	0.043^**^	5.40 ± 3.23	10.80 ± 5.23	0.18	9.92 ± 8.69	9.15 ± 4.60	0.77	0.87
**Biochemical data**										
Hb (mg/dL)	10.12 ± 1.82	12.13 ± 2.81	0.012^**^	13.00 ± 1.27	12.05 ± 1.48	0.65	9.87 ± 1.11	11.20 ± 1.63	0.030^**^	0.79
Urea (mg/dL)	113.92 ± 34.45	99.20 ± 32.75	0.23	125.00 ± 16.97	134.00 ± 12.73	0.18	110.89 ± 46.75	115.67 ± 32.60	0.72	0.42
Uric acid (μmol/L)	5.05 ± 1.60	4.85 ± 1.34	0.65	6.15 ± 1.06	4.80 ±	—	5.80 ± 1.68	5.84 ± 1.38	0.46	0.70
SCr (mg/dL)	10.24 ± 2	10.03 ± 2.9	0.72	11.5 ± 0.91	9.4 ± 4.1	0.65	11.73 ± 2.9	10.40 ± 4.22	0.24	0.17
Pi (mg/dL)	6.57 ± 1.61	5.45 ± 1.12	0.08	6.50 ± 0.00	4.50 ± 2.40	0.18	6.88 ± 1.78	6.58 ± 1.64	0.29	0.49
Cl (mmol/L)	106.43 ± 27.89	98.91 ± 2.95	0.14	98.50 ± 2.12	97.00 ± 1.41	0.65	99.56 ± 2.83	102.00 ± 3.24	0.041^**^	0.42
Vitamin D (nmol/L)	19.79 ± 10.18	28.70 ± 12.65	0.018^**^	35.05 ± 18.74	35.20 ± 12.59	0.65	28.69 ± 13.65	27.18 ± 9.65	0.46	0.82
**Calcium carbonate dose**										
	**1 g**			**2-4 g**			**≥5 g**			
	**n−6**			**n-11**			**n-15**			
	**Baseline**	**Six-months**	**Baseline**	**Six-months**	**Baseline**	**Six-months**	**Baseline**	**Six-months**		
**Oxidants**										
LPO (mM)	16.21 ± 10.81	42.90 ± 31.01	0.11	14.27 ± 2.10	33.33 ± 8.76	0.12	9.71 ± 3.62	27.27 ± 9.04	0.06	0.16
LPO (mM)	16.21 ± 10.81	42.90 ± 31.01	0.11	14.27 ± 2.10	33.33 ± 8.76	0.12	9.71 ± 3.62	27.27 ± 9.04	0.06	0.16
NO (μg/mL)	1,257.37 ± 96.35	1,269.13 ± 165.38	0.75	1,169.39 ± 37.29	1,313.13 ± 42.38	0.022^**^	1,222.37 ± 103.72	1,410.69 ± 172.87	0.008^**^	0.39
8-IP (pg/mL)	53.95 ± 34.28	98.36 ± 65.91	0.10	87.62 ± 15.62	84.42 ± 12.37	0.91	70.70 ± 60.51	75.12 ± 42.07	0.77	0.90
**Markers of oxidative damage to DNA**										
8-OHdG (ng/mL)	7.35 ± 2.37	12.71 ± 2.88	0.10	7.30 ± 1.06	8.47 ± 1.26	0.17	8.49 ± 3.37	8.90 ± 7.03	0.44	0.08
hOGG1 (ng/mL)	5.91 ± 2.96	46.40 ± 26.45	0.28	6.00 ± 1.05	17.71 ± 5.72	0.014^**^	4.66 ± 1.19	15.31 ± 7.5	0.11	0.16
**Antioxidants**										
SOD (U/L)	4.78 ± 3.06	7.08 ± 4.23	0.24	6.31 ± 1.00	8.34 ± 0.96	0.10	4.58 ± 3.04	8.13 ± 4.96	0.017*	0.57
TAC (μM)	0.57 ± 0.17	0.84 ± 0.29	**0.028**	0.65 ± 0.04	0.89 ± 0.07	0.007^**^	0.65 ± 0.22	0.78 ± 0.13	0.011*	0.21
**Pro-inflammatory cytokines**										
IL-6 (pg/mL)	3.82 ± 2.47	3.87 ± 1.36	0.59	3.42 ± 0.42	2.58 ± 0.18	0.08	2.50 ± 0.62	2.60 ± 1.53	0.72	0.41
TNF-α (pg/mL)	2.14 ± 0.55	1.79 ± 0.08	0.28	2.56 ± 0.43	1.99 ± 0.33	0.001^**^	1.92 ± 0.42	1.75 ± 0.38	0.59	0.33
Ferritin (ng/mL)	184.28 ± 146.37	144.16 ± 74.63	0.06	247.69 ± 106.41	320.87 ± 162.70	0.16	176.83 ± 86.49	199.26 ± 104.42	0.11	0.66
CRP (mg/L)	10.30 ± 11.26	20.53 ± 22.33	1.00	5.34 ± 4.82	10.66 ± 6.07	0.008^**^	8.13 ± 9.71	7.87 ± 4.11	0.24	0.57
**Biochemical data**										
Hb (mg/dL)	10.00 ± 0.98	12 ± 0.84	0.08	10.23 ± 2.28	11.51 ± 2.50	0.026^**^	10.74 ± 1.46	11.93 ± 2.26	0.032^**^	0.71
Urea (mg/dL)	79.20 ± 42.32	107 ± 34.73	0.42	126.85 ± 40.02	115.39 ± 31.78	0.08	120.29 ± 36.62	107.75 ± 39.86	0.610	0.19
Uric acid (μmol/L)	4.44 ± 1.41	5.93 ± 0.40	0.11	5.29 ± 1.58	5.13 ± 1.51	0.79	5.22 ± 1.75	5.12 ± 1.56	1.000	0.28
SCr (mg/dL)	8.95 ± 2.51	9.58 ± 3.8	0.75	10.9 ± 3.45	10.31 ± 3.43	0.3	10.42 ± 1.76	9.95 ± 3.16	0.86	0.88
Cholesterol (mg/dL)	182.67 ± 40.30	174.25 ± 28.55	0.46	143.62 ± 30.41	159.63 ± 33.22	0.25	1058.93 ± 901.35	151.11 ± 22.03	0.035^**^	0.36
Pi (mg/dL)	5.48 ± 1.98	5.76 ± 0.82	0.89	6.36 ± 2.36	6.14 ± 1.79	0.22	6.24 ± 1.24	5.40 ± 1.28	0.092	0.90
K (mmol/L)	4.95 ± 0.61	5.06 ± 0.43	1.00	5.37 ± 0.97	5.34 ± 0.61	0.50	5.07 ± 0.61	5.26 ± 0.87	0.47	0.011^***^
HCO_3_(mEq/L)	24.18 ± 2.43	23.68 ± 1.89	0.68	21.70 ± 4.40	22.01 ± 5.12	0.36	24.32 ± 2.88	23.58 ± 1.84	0.20	0.020^**^

*Values are expressed as mean ± standard deviation or standard error of the mean*.

*** *Kruskal-Wallis test*.

** *Wilcoxon rank test. LPO, Lipoperoxides; NO, Nitric Oxide; 8-IP, 8-isoprostanes; 8-OHdG, 8-hydroxy-2'-deoxyguanosine; hOGG1, Oxoguanine glycosylase; SOD, Superoxide dismutase; TAC, Total Antioxidant Capacity; IL-6, Interleukin 6; TNF- α, Tumor necrosis factor alpha; Hb, hemoglobin; SCr, Serum creatinine; K, Potassium; Pi, Phosphate; Cl, Chlorine; CRP, C Reactive Protein; HCO3, Bicarbonate. It was not possible to measure the glomerular filtration rate since its value is <10 mL/min/1.73 m^2^. Data in bold are statistically significant p-values*.

### Hemodialysis and Calcium Carbonate Dose

Patients who ingested calcium carbonate in daily doses of 2–4 g showed an increase in NO, 1,313.13 ± 42.38 μg/ml (*p* = 0.043), and even those who ingested ≥ 5 g per day had increased NO levels at 6 months of follow-up, 1,410.69 ± 172.87 μg/ml (*p* = 0.008). The DNA repair enzyme was significantly increased at the final determination in patients who ingested 2–4 g of calcium carbonate, 16.23 ± 5.78 pg/ml (*p* = 0.009).

The activity of the antioxidant enzyme SOD was significantly increased in those who ingested ≥ 5 g in the final determination of the study, 8.13 ± 4.96 U/L (*p* = 0.017). TAC was determined to be significantly increased at 6 months of follow-up in those who ingested 1 (0.84 ± 0.29 μM, *p* = 0.028), 2–4, (0.89 ± 0.07 μM, *p* = 0.007), and ≥ 5 g (0.7 8 ± 0.13 μM (*p* = 0.011).

The expression of TNF-α decreased significantly in the final determination of those who ingested 2–4 g, 1.99 ± 0.33 pg/ml (*p* = 0.001). Hemoglobin improved with the doses of 2–4 (11.51 ± 2.5 mg/dl) and ≥5 g (11.93 ± 2.26 mg/dl, *p* =0.032). CRP was significantly increased in patients who ingested 2–4 g at 6-month follow-up 10.66 ± 6.07 mg/L (*p* = 0.008). PTH was significantly increased in patients who ingested 1 g of calcium carbonate, 652.86 ± 217.31 pg/ml (*p* = 0.043) (Table 4).

### Correlation Between Clinical Data and Markers of Inflammation and OS

Table 5 shows the moderate negative correlations found between SOD and PTH (rho = −0.052, *p* = 0.035), TAC and CRP (rho = −0.58, *p* = 0.017), for sevelamer hydrochloride. For calcium carbonate, moderate negative correlations were found for hOGG1 and HCO3 (rho = −0.68, *p* = 0.05), and LPO and pH (rho = −0.67, *p* = 0.035). Those with high correlations included 8-OHdG and urea (rho = −0.7, *p* = 0.024), and hOGG1 and K (rho = 0.7, *p* = 0.038) (Table 5).

**Table 5:** Correlation between oxidative stress markers and clinical parameters.

**Redox biomarker**	**Clinical parameter**	**Sevelamer hydrochloride**		**Calcium carbonate**	
		**rho**	***p***	**rho**	***p***
LPO	pH	0.53	**0.025**	−0.67	**0.035**
8-IP	Cl	0.56	**0.046**	−0.04	0.91
8-IP	HCO_3_	0.58	**0.049**	−0.21	**0.056**
8-OHdG	Urea	−0.14	0.67	−0.7	**0.024**
hOGG	K	−0.01	0.98	0.7	**0.038**
hOGG1	Urea	0.65	**0.016**	0.49	**0.09**
hOGG	Cholesterol	−0.35	0.15	−0.72	**0.043**
hOGG	HCO_3_	−0.38	0.25	−0.68	**0.05**
SOD	PTH	−0.52	**0.035**	0.61	**0.15**
TAC	CRP	−0.58	**0.017**	−0.1	0.87
TAC	Cl	0.43	**0.046**	0.2	0.58
IL−6	TSAT	−0.67	**0.0.13**	−0.14	0.76
IL−6	Vit D	0.2	0.65	1	**<0.01**
IL−6	HCO_3_	−0.65	**0.022**	0.12	0.75

*Data in bold are statistically significant p-values*.

## Discussion

**Figure 2:**
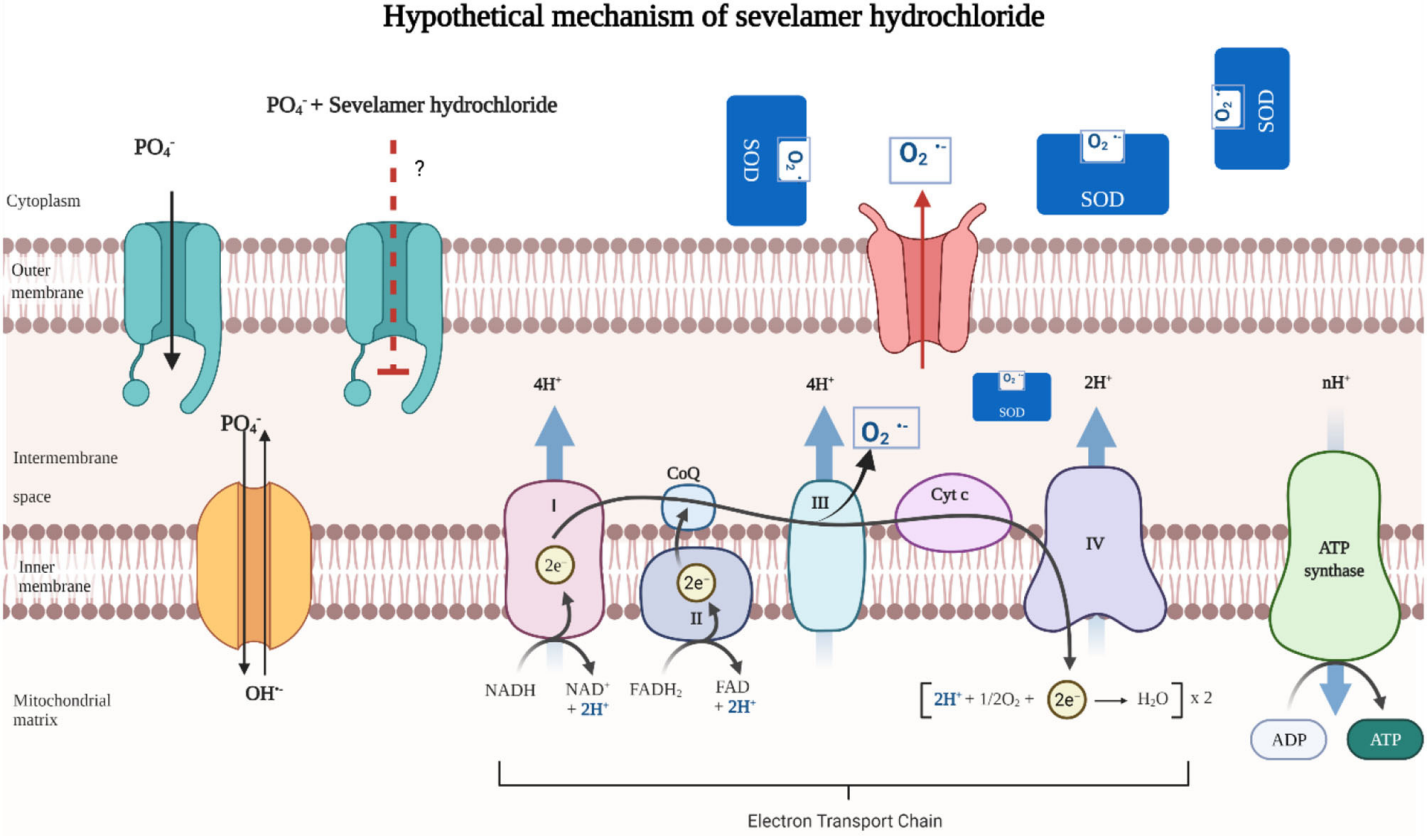
Hypothetical mechanism of sevelamer hydrochloride in oxidative stress (OS). Increase in extracellular Pi causes mitochondrial OS conditioned by mitochondrial hyperpolarization produced by a phosphate-hydroxyl exchange. Hyperpolarization stimulates the electron transport chain and increases the concentration of superoxide radicals by complex III. The binding of Pi to sevelamer hydrochloride could explain the antioxidant mechanism by reducing mitochondrial OS (20).

Most patients on HD are treated with phosphate binders to reduce serum levels and intestinal absorption of this mineral (15). It is known that dietary Pi restriction is not sufficient to maintain serum concentration within recommended levels. The management of hyperphosphatemia includes various chelators, such as sevelamer hydrochloride and calcium carbonate (16). Sevelamer has been observed in previously published research to reduce the absorption of advanced glycation products (AGE), bacterial toxins, and bile acids, suggesting that this mechanism reduces inflammatory, oxidative, and atherogenic stimuli in addition to its direct action of reducing serum Pi (17). Recently, some antioxidant and anti-inflammatory pleiotropic effects of sevelamer hydrochloride not associated with Pi depletion have been described (18). Elevated extracellular Pi causes mitochondrial OS related to mitochondrial hyperpolarization (19). The binding of Pi to sevelamer hydrochloride could explain the antioxidant mechanism by reducing mitochondrial OS. In this clinical study, the influence of sevelamer hydrochloride and calcium carbonate on markers of inflammation, oxidants, antioxidants, and oxidative DNA damage in patients on HD with 6 months of follow-up was evaluated (Figure 2).

Calcium carbonate is the first-line treatment for lowering Pi in ESRD. However, its main effect is to avoid significant hyperphosphatemia without normalizing Pi serum levels. Adequate adherence to treatment with calcium carbonate avoids the uncontrolled rise of hyperphosphatemia (21).

Lipoperoxidation produces increased OS, leading to the formation of molecules such as aldehydes (malondialdehyde), and whose measurement is used to determine OS (22). HD can remove water-soluble, low molecular-weight LPO products. However, the HD process can also favor the increase in LPO depending on the time spent in the HD program (23). The prolonged stay of a patient in renal replacement therapy is considered a contributing factor to increasing inflammation and OS (24). In this study, the results at 6-months follow-up showed an increase in LPO in patients treated with the 3,200 mg dose of sevelamer hydrochloride.

NO is a molecule with a wide variety of fundamental physiological functions, such as maintenance of muscle tone. It is an intracellular messenger, a cytotoxic agent, and has a primordial effect on the vascular endothelium. NO has specific functions in the kidney, regulating hemodynamics, salt and water reabsorption, renin secretion, and tubule-glomerular feedback. Its bioavailability abnormalities are causally related to various cardiovascular and renal disorders (20). In patients with ESRD, there could be decreased NO synthesis due to kidney damage (25). The disturbed balance of NO is related to the imbalance of endothelin-1 (ET-1) (26). NO is involved in the pathogenesis of hypertension and rebound hypotension during the HD process (27). The female gender tends to have better kidney function and higher NO concentration (as usual) due to the action of estrogens (28). According to previous studies, the bioavailability of NO in patients with DM is low (29, 30). Endothelial dysfunction is a key step in the development of atherosclerosis in HD. Endothelial dysfunction has been attributed to altered NO bioactivity and increased formation of oxygen-derived free radicals. The underlying mechanisms of the altered bioavailability of NO in patients on HD are not fully understood. However, the activation of cytokines during HD can increase NO production (31, 32). The increase in NO levels in the patients who ingested sevelamer hydrochloride could suggest that they were under nitrosative stress in the final follow-up. This phenomenon occurred in patients who ingested doses of 1,600 and 3,200 mg. In the group that ingested calcium carbonate, NO levels also increased in the determination at 6 months of follow-up and when the patients ingested amounts >2 g, suggesting that they were also under nitrosative stress.

8-Hydroxy-2-deoxyguanosine (8-OHdG) is a marker that determines oxidative damage to DNA (33, 34). OS determined by the 8-OHdG marker is an independent predictor of all-cause mortality and morbidities in patients on HD (35, 36). Previously, it has been found that the oxidative damage to DNA evaluated with the marker 8-OHdG in peripheral blood lymphocytes increased more in patients on chronic HD than in non-dialyzed patients (37). On the other hand, sevelamer hydrochloride has previously been reported to have the ability to reduce oxidative damage to genetic material (38). The authors of a recent study reported the potential effect of sevelamer treatment on inflammation and possible oxidative RNA modifications (39). The results obtained at 6 months of follow-up of the patients on HD included in these studies who ingested sevelamer hydrochloride showed a significant increase in the oxidative DNA damage marker different from previously reported.

Repair of oxidative DNA damage is essential to maintain the integrity of genetic material, prevent mutagenesis, and decrease damage caused by reactive oxygen species. The hOGG1 enzyme is responsible for identifying and repairing oxidative damage to DNA through base cleavage mechanisms (40). High levels of pro-inflammatory cytokines and certain oxidants can decrease the activity of the DNA repair enzyme, especially in patients with ESRD (41, 42). The authors of a recent publication reported the downregulation of DNA repair enzymes in patients dependent on the type of peritoneal transport (43). Significant increase in oxidative DNA damage repair enzyme levels in patients treated with sevelamer hydrochloride, at 6 months of follow-up, is notable, especially in those who ingested 1,600 mg of the phosphate binder. Given this finding, we could hypothesize that the significant increase in DNA repair enzyme in the sevelamer hydrochloride group of patients on HD could be compensating for the increased oxidative damage marker in DNA. In contrast, no modification of the oxidative DNA damage marker or DNA repair enzyme was observed in those who ingested calcium carbonate.

The SOD enzyme is the first antioxidant defense of the body. The dismutation of the superoxide anion characterizes the SOD enzyme into oxygen and hydrogen peroxide (44). Increased activity of the antioxidant enzyme SOD in patients on HD with or without hyperglycemic status is well-documented. Increased SOD activity is considered an indicator of future vascular complications in patients on HD (45, 46). Elevated extracellular Pi causes mitochondrial OS related to mitochondrial hyperpolarization (20). The binding of Pi to sevelamer hydrochloride could explain the antioxidant mechanism by reducing mitochondrial OS. In this study, a significant increase in the activity of the SOD enzyme was found in the groups treated with sevelamer hydrochloride and with calcium carbonate in the determination at 6 months of follow-up. The increase in the activity of the SOD enzyme was notorious in those who ingested 3,200 mg of sevelamer hydrochloride (Figure 2).

Total antioxidant capacity determines the sum of endogenous and exogenous antioxidants (47). Advanced glycation end (AGE) products are excreted in the urine of subjects with normal renal function. However, in patients with ESRD who require HD, AGE products accumulate in the body from insufficient urinary excretion and limited clearance during dialysis (48). Treatment with sevelamer hydrochloride has previously been reported to reduce systemic and cellular AGE levels by restoring innate antioxidant defenses, improving inflammatory status, and reducing chronic OS (49, 50). At the end of the follow-up period of this study, SOD enzyme activity and TAC levels were found to be increased in the groups treated with sevelamer hydrochloride and calcium carbonate. These findings could suggest an attempt to compensate for the increase in oxidant molecules LPO and NO.

Mediators that orchestrate inflammatory response are cytokines. Among the cytokines, IL-6, and TNF-α have profound effects on the pro-inflammatory process in patients undergoing HD (51). According to our results, sevelamer hydrochloride showed an anti-inflammatory effect (52). Sevelamer hydrochloride can bind the endotoxins present in the intestinal lumen. In this way, the negatively charged lipid. Endotoxins are a powerful stimulus with the ability to activate the innate immune system that favors the transcription and production of pro-inflammatory cytokines. A part of endotoxins binds to sevelamer. In this way, sevelamer hydrochloride could exert its anti-inflammatory effect (53, 54). IL-6 decreased significantly in the determination at 6 months of follow-up in patients who ingested calcium carbonate and those who ingested 1,600 mg of sevelamer hydrochloride. TNF-α showed downregulation at 6 months of follow-up in the sevelamer hydrochloride and calcium carbonate groups. TNF-α decreased its levels primarily in those who ingested 3,200 mg of sevelamer hydrochloride and in those who ingested 2–4 g of calcium carbonate.

**Figure 3:**
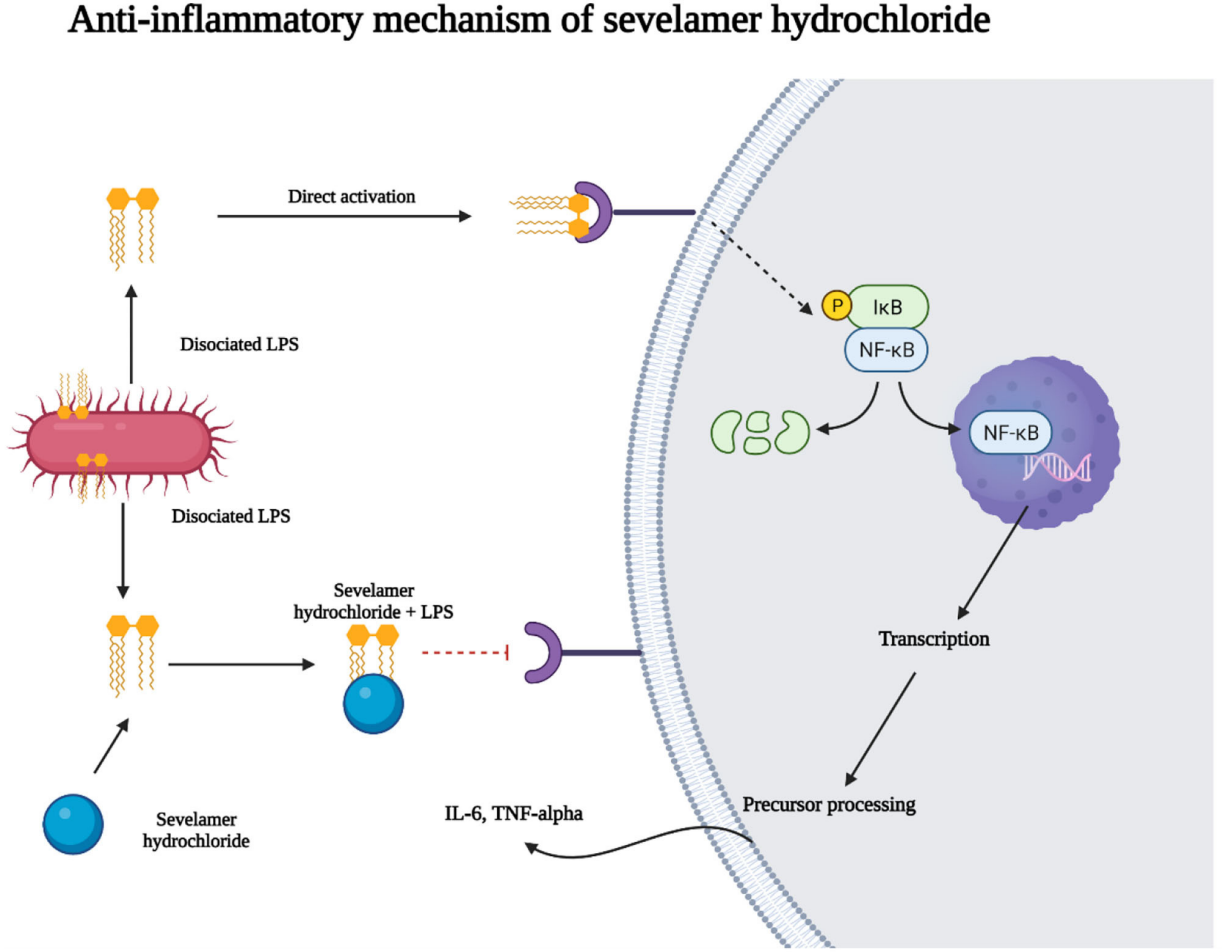
Anti-inflammatory of sevelamer hydrochloride. Sevelamer hydrochloride can bind to endotoxins present in the intestinal lumen. In this way, the negatively charged lipid A portion of endotoxins binds to sevelamer. Endotoxins are a component of the cell wall of gram-negative bacteria and are a potent stimulus for activating the innate immune system that leads to the transcription of pro-inflammatory cytokines. In this way, sevelamer hydrochloride could exert its anti-inflammatory effect.

Sevelamer hydrochloride (more significant effect) and calcium carbonate showed anti-inflammatory and antioxidant effects independent of the phosphate inhibitory effect as previously reported (55, 56). Inflammatory markers, such as CRP, progressively increase with deterioration in renal function (57). Available data, such as CRP, TNF-α, IL-6, and arteriovenous fistula dysfunction, demonstrated that these systemic inflammatory markers were elevated in patients on HD (58). CRP was found to be increased in the final determination at the follow-up in those who ingested calcium carbonate, 2–4 g of calcium carbonate, and 1,600 mg of sevelamer hydrochloride. Alteration of serum ferritin may be a determining factor in mortality in adults on HD, and high ferritin levels lead to high mortality (59). The alteration of serum ferritin can be a determining factor in mortality in adults on HD. At the end of the follow-up of the patients who ingested sevelamer hydrochloride, ferritin levels decreased. In patients who ingested 1,600 and 3,200 mg of sevelamer hydrochloride, ferritin levels were decreased (Figure 3).

## Conclusions

The pleiotropic antioxidant and anti-inflammatory effects of sevelamer hydrochloride were fundamental in the results of this research study. OS was manifested by a significant increase in LPO and NO levels in the final determination at 6 months of follow-up in the sevelamer hydrochloride group and with the intake of 1,600 and 3,200 mg. The increase in NO at the end of the follow-up favored OS in the calcium carbonate group when they ingested doses of 2–4 and >5 g. OS is not only explained by the intake of Pi binders. ESRD and HD could play an essentially active role in their presence. As a probable compensatory mechanism for the increase in OS markers, levels of antioxidant enzymes were found to be increased at 6 months of follow-up. Increase in the SOD enzyme and TAC activity was observed in the sevelamer hydrochloride group and calcium carbonate groups in the final determination at the follow-up. A more significant increase in SOD activity was observed when the patients ingested 1,600 and 3,200 mg of sevelamer hydrochloride. In the calcium carbonate group, the increase in TAC was observed when they ingested from 1, 2–4, and more than 5 g. The behavior of pro-inflammatory cytokines was downregulated; primarily, TNF-α decreased its levels in the final determination at six months of follow-up in the groups that ingested sevelamer hydrochloride and calcium carbonate. Decrease in TNF-α is predominant in those who ingested 2–4 g of calcium carbonate or 3,200 mg of sevelamer hydrochloride. IL-6 decreased in those who ingested 1,600 mg of sevelamer hydrochloride and in the final determination of the calcium carbonate group, suggesting that both phosphate inhibitors have anti-inflammatory effects. Ferritin levels decreased significantly at 6 months of follow-up in the sevelamer hydrochloride group. This phenomenon was predominantly observed when they ingested the 1,600 and 3,200 mg doses, suggesting an anti-inflammatory improvement.

Contrary to the sevelamer hydrochloride, those who ingested calcium carbonate did not show changes in their levels. CRP levels increased in the final determination of the calcium carbonate group. The beneficial influence of sevelamer hydrochloride on pro-inflammatory cytokines and OS markers could be essential in managing phosphate binders in patients on HD by offering advantages in morbidity. However, the mechanism of action of sevelamer hydrochloride in OS is not fully understood. Long-term studies with a larger number of patients are required to know all the benefits that they can provide to patients with ESRD.

### Study Limitations

The limitations of this study are based on the small number of patients in the HD program and the short follow-up time. We consider it worthwhile to design another stratified analysis that considers several phosphorus inhibitors for different follow-up times.

### Study Strengths

The study offers a broader view on the simple use of two phosphate binders, the widely known calcium carbonate, and sevelamer hydrochloride, with probable anti-inflammatory pleiotropic effects. The information reported on the impact of sevelamer hydrochloride on the repair of oxidative DNA damage is scarce. In this study, we found the overexpression of the DNA repair enzyme possibly compensating for the significant increase in the oxidative DNA damage marker in patients on HD. This study provides new information on the possible regulatory effects of sevelamer hydrochloride on DNA repair.

## Data Availability Statement

The data raw that support the conclusions of this article will be made available by the authors, with prior authorization from the Research and Ethics Committee.

## Ethics Statement

The study was followed under the ethical principles for medical research in human beings stipulated in the declaration of Helsinki 64th General Assembly, Fortaleza Brazil, October 2013, and the Standards of Good Clinical Practice according to the guidelines of the International Conference on Harmonization. Under the General Health Law's provisions following the Regulations of the General Health Law on Research for Health Article 17 of Mexico, the study corresponds to category III. All the patients signed the Low Information Consent in the presence of witnesses. The study was evaluated and approved by the Local Ethics and Research Committee at the CentroMédico Nacional de Occidente, Instituto Mexicano del Seguro Social (R-2021-1301-063).

## Author Contributions

ED-D, AG-S, AGM-D, and JC-G: conception and design. CP-N, JA-S, BJ-M, AB-L, and ER-C: acquisition, analysis, and interpretation of data. AG-S, ED-D, and AGM-D: drafting the article and revising it critically for important intellectual content. ED-D, AG-S, AGM-D, JC-G, CP-N, JA-S, BJ-M, AB-L, and ER-C: final approval of the version to be published. All authors contributed to the article and approved the submitted version.

## Conflict of Interest

The authors declare that the research was conducted in the absence of any commercial or financial relationships that could be construed as a potential conflict of interest.

## Publisher's Note

All claims expressed in this article are solely those of the authors and do not necessarily represent those of their affiliated organizations, or those of the publisher, the editors and the reviewers. Any product that may be evaluated in this article, or claim that may be made by its manufacturer, is not guaranteed or endorsed by the publisher.
